# NeuRec: Incorporating Interpatient prior to Sparse-View Image Reconstruction for Neurorehabilitation

**DOI:** 10.1155/2022/5426643

**Published:** 2022-05-09

**Authors:** Cong Liu, Qingbin Wang, Jing Zhang

**Affiliations:** ^1^Faculty of Business Information, Shanghai Business School, Shanghai 200235, China; ^2^The Affiliated Changzhou No. 2 People's Hospital of Nanjing Medical University, Changzhou 213003, China; ^3^Center of Medical Physics, Nanjing Medical University, Changzhou 213003, China; ^4^Ruijin Hospital, Shanghai Jiao Tong University, Shanghai 200031, China

## Abstract

Medical imaging technologies such as computed tomography (CT) and magnetic resonance imaging (MRI) imaging are indispensable for contemporary neurorehabilitation diagnostics, intervention, and monitoring. It would be desirable to reconstruct images from sparse measurements to reduce the ionizing radiation and motion artifacts. Although recent coordinate-based representation methods have shown promise advances for sparse-view reconstruction, they overfit a single MLP on a single patient. In this work, we generalize it across many patients by incorporating an interpatient prior into the ill-posed inverse/reconstruction problem, which is the missing ingredient in the previous works. The experiment demonstrates that our method significantly improves image quality over the state-of-the-art both qualitatively and quantitatively. Thus, our method provides a powerful and principled means to deal with the measurement-scarce problem.

## 1. Introduction

The majority of patients that enter neurorehabilitation therapies will have undergone some kind of medical imaging [1]. Computed tomography (CT) and magnetic resonance imaging (MRI) are the two most common modalities for neuroimaging. CT scans of the brain are much faster than MRI making it the ideal choice in cases of trauma and other acute neurological emergencies, where a rapid decision regarding appropriate medical management is imperative. The soft-tissue details generated by a CT are usually acceptable for clinicians to assess the acute damage, while MRI has a much greater range of available soft-tissue contrast. Although their broad application in daily rehabilitation practice, CT, and MRI have had their own weakness since their early days, CT imaging involves the use of x-rays, which are a form of ionizing radiation. Exposure to ionizing radiation is known to increase the risk of cancer. MRI requires a longer scan time than CT to collect sufficient data to form an image thereby being sensitive to excessive patient motion during the actively scanning. The effects of motion include blurring and ghosting in the resulting image [2]. One possible method of addressing these issues is to reconstruct CT or MRI from partial measurements such as sparse-view projections/sinograms and low-resolution *k*-spaces. The sparse-view restriction reduces the requirements of the number of measurements thereby lowering the ionizing radiation for patients and suppressing motion artifacts in clinical MRI.

However, reconstructing images from limited data, as well as the ill-posed inverse problem, is very challenging to be solved. For example, for sparse-view sinograms, the corresponding projection matrix or Radon transform has more columns than rows. It can be shown that null vectors always exist for this underdetermined linear system, which means the solution for this system is not unique; more than one image may yield the same measurement. We can obtain a reasonable solution from the least-square minimum norm, but the solution is usually smoothed version of the original because the null vectors that are not measurable are disregarded. To obtain a better reconstruction, the conventional methods [3–6] add the prior information of the unknown images such as sparse prior (e.g., total variation) and redundant prior (nonlocal mean) to the null space. These methods have been shown to achieve accurate recovery of the real images under some circumstances. Machine learning methods [7–9] especially the convolutional neural networks (CNNs) have recently emerged as the state of the art for solving the inverse problem though clinical-sized applications have yet to be demonstrated.

The CNN-based reconstruction methods can be categorized into three main directions. The first one is the learned postprocessing methods [10] where a learned operator is applied to the images that are reconstructed by conventional methods such as filtered back-projection (FBP) [11]. This operator performs the enhancement, denoising, and sharpness to improve the image quality. This type of method is relatively easy to be implemented because the physics principles are not involved. The second method is to learn the prior and use this prior in the classical iterative reconstruction formulation [12]. This kind of priors is learned from data, rather hand-designed, based on a large training patient dataset. The learned priors introduce the data priors to constraint the null space showing promise results though clinical-sized applications have yet to be demonstrated. The final method directly learns the reconstruction from the partial measurements in a data-driven style [13]. The partial measurements are directly mapped to the images by supervised training on a large dataset consisting of the pairs of measurements and images. Although these methods show outstanding performance for medical image reconstruction, they also have some shortages. For example, the requirement of a large-scale training dataset may prevent the CNNs from generalizing to the unseen view angles. Another issue is their memory-hungry feature inheriting from the dense voxel-based representation. While dense voxel-based representations are fast to query, they are memory inefficient, and 3D CNNs, potentially operating on these volumes, are computationally heavy.

To overcome the aforementioned problem, we propose neuRec (neural reconstruction) framework which is based on the recent implicit neural representation [14]. Instead of storing human tissues using voxels, it defines a coordinate-based multilayer perceptron (MLP) to approximate the quantities of interest, e.g., electronic density in CT. The MLP maps continuous coordinates to voxel values, which is in contrast to conventional CNNs methods that propagate and backpropagate the 3D voxels tensor through the entire network. The coordinate-based MLP representation offers potential compact representation in terms of memory efficiency. Moreover, theoretically, it presents voxels at infinite resolution because continuous coordinate is used as the input of the network. The continuous assumption is also a useful characteristic for the sparse-view reconstruction, which enforces spatial coherence to the null space. The spatially nearby voxels are constraint to be associated with the same value implicating human tissues change slowly over space. The spatial coherence prior can also be combined with other priors for medical image reconstruction as shown in recent works [15, 16]. However, only interpatient priors such as previous CT are employed in these works. Our neuRec differs from them in that the interpatient prior is exploited for the patient in hand.

## 2. Methods

### 2.1. NeuRec

**Figure 1:**
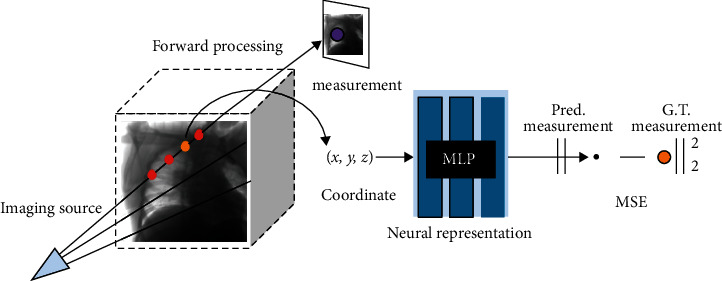
A schematic diagram of the neuRec.

As illustrated in Figure 1, we aim to learn a coordinate-based representation *f*_*θ*_ : X⟶*Y* where the voxel coordinate *x* ∈ *X* is mapped to voxel value *y* ∈ *Y*, and the mapping is parameterized with  *θ*. Given the limited number of physic measurements *z* and their corresponding geometry specifics, we can approximate the measurements using linear perturbation theory with forwarding process *z* = *Fy*, where *F*  is the linearized measurement operator or Jacobian. If the measurement is performed *M* times and the image has *N* voxels, then *F* has the shape of *M* × *N*. For sparse-view measurements, the transform  *F* is a short, fat matrix, i.e., *M* ≪ *N*.

The reconstruction or inverse problem is to the unknown image *y* when *z* and *F* are given. To solve *y*, we turn to an optimal mapping *f*_*θ*_^∗^ that can be obtained by the lowest average mean-squared distance to the true measurement *z*:

(1) $$\begin{array}{c} \;{\text{argmin}}_{\theta }\;E_{\left(x,z\right)\sim \mu }\;\left[{\left\|F\left(\left(f_{\theta }\left(x\right)\right)\right.-z\right\|}_{2}^{2}\right], \end{array}$$

where *f*_*θ*_(*x*) predicts the voxel values that are then forwarded to predicting measurements *F*(*f*_*θ*_(*x*)) and are compared with the ground truth measurements *z*. Here, the expectation is taken w.r.t the joint probability distribution *μ* of random variables (*x*, *z*). Since we generally do not have access to the true distribution *μ*, we then replace the expectation in Equation (1) with its empirical estimator

(2) $$\begin{array}{c} \theta^{*}={\text{argmin}}_{\theta }\frac{1}{K}\overset{K}{\sum }\left[{\left\|F\left(f_{\theta }\left(x_{k}\right)\right)-z_{k}\right\|}_{2}^{2}\right], \end{array}$$

where  *k* denotes the individual sample for a patient and *K* is the number of all samples. After obtaining the optimal *f*_*θ*_, we can easily compute the corresponding optimal voxel values with the mapping *f*_*θ*_^∗^(*x*). As we mentioned before, the coordinate-based representation has a build-in spatial coherence prior. This can be explained by noticing Equation (2), where the coordinate *x* is assumed to follow a continuous distribution though the discrete samples are drawn from this distribution to train the *f*_*θ*_.

To further constraint the sparse-view inverse problem, we introduce the interpatient priors in the next. To put it simply, we train the coordinate-based representation *f*_*θ*_ on a large patient dataset such that the resulting *θ* is set at an appropriate point where the pretrained model can generalize to the patient in hand. To adapt the coordinate-based representation *f*_*θ*_ to variable voxel values and positions, we adopt the Generative Latent Optimization (GLO) [17] in which each patient is assigned a corresponding low-dimensional latent vector that encodes the patient-dependent specifics. We then change the input of the MLP mapping *f*_*θ*_(*x*_*k*_) with a patient-dependent variable *e*_*i*_ to have

(3) $$\begin{array}{c} f_{\theta ,e_{i}}\left(x_{k}^{i}\right), \end{array}$$

where *i* denotes the patient index and *k* index the training samples for the patient *i*. To reconstruct the voxels for a patient *i*, the latent vector *e*_*i*_ is also optimized so that the prediction from the MLP *f*_*θ*,*e*_*i*__(*x*_*k*_^*i*^) matches the measurement *z*_*k*_^*i*^ of this patient. With the low-dimensional latent vector, we condition the coordinate-based representation *f* on a patient to enable the network to explain certain voxel variations derived from this patient. The network parameter *θ* is trained and shared among all patients and serves as an initial weight for a new patient. Moreover, the shared parameter *θ* acts as a strong prior that encodes the common human anatomy structures, which enables both better generalization and faster convergence during optimization.

Autoencoders or encoder-decoder networks are widely used for representation learning as their bottleneck features tend to form natural latent variable representations [18, 19]. The Generative Latent Optimization used here is different from the autoencoders in that only the decoder part of the latter is retained for training and inference. The encoder part of the autoencoders is dropped as it may waste computational resources. Without the encoder, the latent space can still emerge as we search for an optimal latent variable representation during the training [20].

We follow the classical ResNets [21] to implement our MLP network except by using feedforward layers in place of convolutional layers, i.e., residual multilayer perceptron (ResidualMLP). The inputs are fed to a sequence of MLP blocks to produce output embeddings. Similar to ResNets, each block is paralleled with a skip-connection [21] to improve the convergence. We implemented ResidualMLP with six residual blocks and condition the network on the latent vector of dimension 16. The shortcut connections are used to connect the adjacent blocks. Each block consists of the affine layer defined by the weight matrix and the biases applied on its input, followed by a sine nonlinearity applied to each component of the resulting vector. We used 256 neurons for each hidden layer. To model the variation across the patients, a small MLP is constructed with 2 affine layers of 256 neurons so the dimension of the resulting feature vector is the same as the main network. We then add the resulting feature vector to each block's outputs to adapt the main network for the patient-specific. The output dimension of the last layer is 1.

### 2.2. Data

We used two datasets in this study. Dataset 1 contains a set of MRI data volumes produced by an MRI simulator [22]. These data are usually used by the neuroimaging study to evaluate the performance of various image analysis methods in a setting where the truth is known. Dataset 2 consists of customized versions of Shepp-Logan phantom [23]. The radians, angles, and positions of 3D ellipses are generated randomly. The Shepp-Logan phantom is often used in CT reconstruction to evaluate the performance of the reconstruction algorithms.

### 2.3. Implementation Details

We first train the network and the latent vector with all training samples from two datasets in the proposed neuRec way, then finetune the network from the pretrained checkpoint on the remaining test patients to evaluate the performance. The latent vector is initiated randomly and not optimized during the finetune. Two NVIDIA TITAN 3090 GPUs and PyTorch [24] deep learning framework are used to develop codes. For training the model, the measurements are centered and normalized by the mean and standard deviation across the training samples. We implement the details suggested in literature [25] to boost the performance, i.e., the Adam optimizer is used with a custom learning rate schedule that warms up linearly from 0 to 1*e*-4 for the first 100 iterations, then decays proportional to the square root of the step count. The training takes about 17 hours due to the large patient samples, and the inference takes only several milliseconds due to the simplicity of the network.

### 2.4. Quality Evaluation Metrics

For reconstructions we compute the mean squared error (MSE), structured similarity (SSIM) index, and peak signal-to-noise ratio (PSNR) values with respect to the ground truth image (higher is better).

## 3. Results and Discussion

### 3.1. MRI Reconstruction

In order to verify the quality of the new method in MRI reconstruction, it is compared with the other four methods: filtered back projection (FBP) [26], simultaneous algebraic reconstruction technique (SART) [27], total variation (TV), and nonlocal mean (NLM) priors solved by the primal-dual algorithm (TV-NLM-PDHG) [28], and Deep Prior [29]. The FBP reconstruction is used as the representation of classical analysis methods. We use the Hann filter for its filter stage. SART is superior to the standard algebraic reconstruction technique and is useful in cases when the projection data is limited. The TV corresponds to the local structure prior, while NLM aims to regularize the image statistics at a global level. PDHG iterative reconstruction is performed using 1000 iterations of the classical PDHG algorithm. Deep prior is a CNN-based prior for the inverse problem which captures the low-level image statistics in the structure of the network.

**Figure 2:**
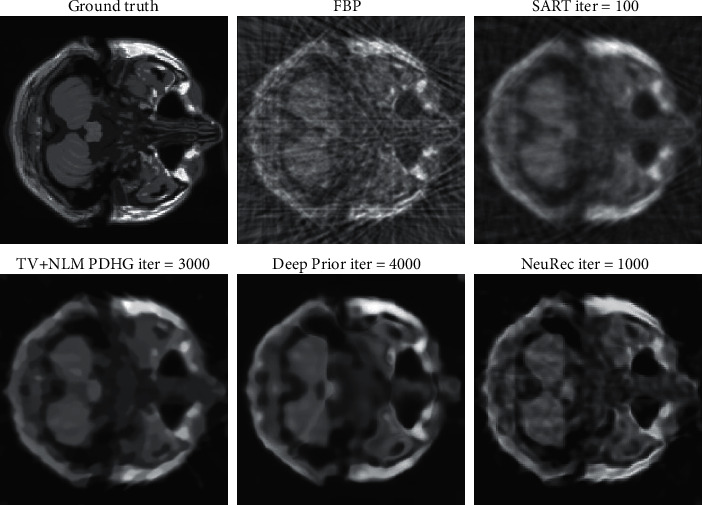
MRI reconstruction comparison.

As illustrated in Figure 2, the classical FBP method performs worst because the analytical method assumes a complete measurement is available which prevents it from a plausible reconstruction. The SART method outperforms the FBP qualitatively, as it suppresses the artifices effectively and produces a clearer image. Compared with the last two methods, the TV-NLM-PDHG algorithm produces a better reconstruction because the local- and global-prior are introduced to make up the null space. Finally, our neuRec method shows unprecedented qualitative results as high-frequency features such as the hippocampus are distinctly observed. The quantitative results in Table 1 also suggest a significant increase of PSNR and SSIM values is obtained with our neuRec.

### 3.2. CT Reconstruction

**Figure 3:**
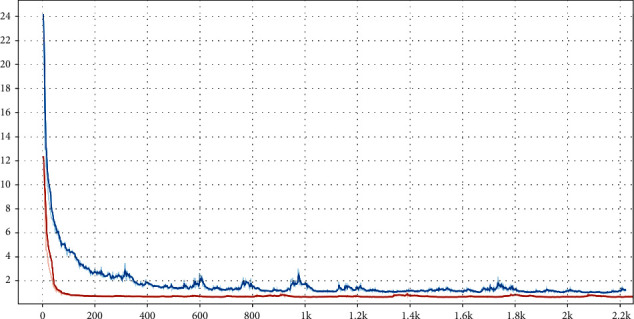
Ablation study. The MSE of neuRec with interpatient (red) and without interpatient prior (blue) is compared.

**Figure 4:**
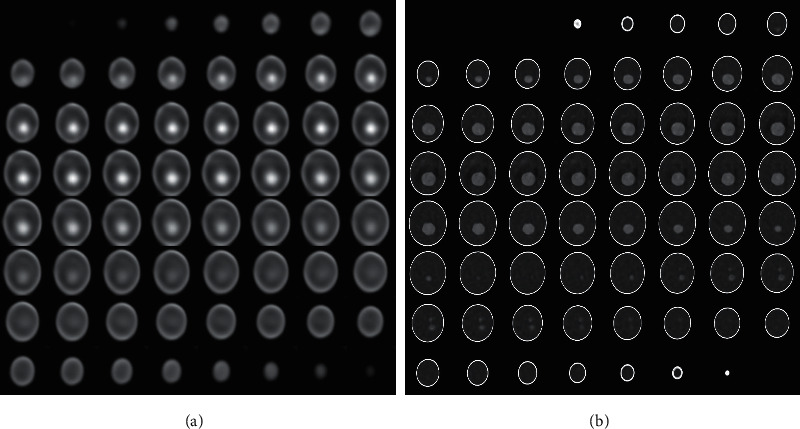
Qualitative ablation study. Reconstruction of neuRec without interpatient (a) and with interpatient (b).

To demonstrate the impact of interpatient prior, we carry on the experiments with the prior and without the prior for CT sparse-view reconstruction. Only 10 sinograms of the 3D Shepp-Logan phantom are used to push the neuRec to its limitation.  Figure 3 shows the MSE with respect to the test iteration for neuRec with and without the interpatient prior. From the curves, we find neuRec with interpatient prior enables faster and more accurate reconstruction than neuRec without interpatient prior. To verify this conclusion, we further plot the 100-iteration intermediate results in Figure 4. As we can see from the figure, neuRec with interpatient prior achieves a much better reconstruction than neuRec without interpatient prior, even though only 10 sinograms are used. The sparse-view reconstruction, i.e., the ill-posed inverse problem is very challenging to be solved. As illustrated in the figure, the results produced by NeuRec without interpatient prior tend to be smoothed because the least-square minimum in Equation (2) estimates the average of the sparse samples. On the contrary, with interpatient prior, the network parameter is trained and shared among all patients and serves as an initial weight for a new patient. The shared parameter acts as a strong prior that encodes the common human anatomy structures, which enables both better generalization and faster convergence during optimization.

## 4. Conclusions

This study proposes and evaluates a novel deep learning-based method neuRec for the sparse-view reconstruction of medical images, such as MRI and CT. It solves this ill-posed inverse problem through a coordinate-based neural network representation and the introduction of interpatient prior that is not exploited in other works. We demonstrate its performance on two datasets where it outperformed several state-of-the-art reconstruction methods. Although we presented results for CT and MRI reconstruction from sinograms and *k*-spaces, neuRec can be used whenever the rendering or measurement process is differentiable. A possible line of research is to investigate interpatient priors for solving the more aggressive inverse problem.

## Figures and Tables

**Table 1:** Comparison of the neuRec in MRI reconstruction with state-of-the-art methods.

Metrics	neuRec	FBP	SART	TV-NLM-PDHG	Deep prior
SSIM	0.873	0.201	0.623	0.718	0.843
PSNR	31.6	15.3	22.6	25.3	27.6

## Data Availability

Research data are stored in an institutional repository and will be shared upon request to the corresponding author.
